# Supplementary material for: “Around the clock”: Exploring health care professionals’ experience of discharge of older people during out of hours from the emergency department: A qualitative study
Source: PLoS One. 2025 Aug 22;20(8):e0313968. doi: 10.1371/journal.pone.0313968 (PMC12373235; doi:10.1371/journal.pone.0313968)
Supplement: S1 Appendix — Older people out of hours discharge from the emergency department research study. (PDF) [file pone.0313968.s001.pdf]

S1 Appendix 1

Interview Schedule for Health Care Professionals in the Emergency Department

This phase of the research study will comprise interviewing senior health care professionals in the emergency department regarding the discharge of older people from the ED. These interviews will be conducted via video conferencing or telephone over a 50–60-minute timeframe.

Introduction

Introduce self

Give an outline of the interview given

Explain the purpose of the study

Questions for the semi-structured interview with senior healthcare professionals in the emergency department

Role of the participant:

Confirm the role of the participant i.e., CNM3, Consultant, etc.

How long have you been in position?

Can you tell me about your role in the ED? E.g., in an office, on the floor partaking in clinical practice; if and how this has changed during the COVID-19 pandemic?

In your role in the ED how actively are you involved in discharging older people from the ED?

Definitions:

In your ED, what is considered as out of hours?

What is considered as the definition for an older person?

Explain that for the purpose of this study, the older person will be defined as 75 years or older.

Supports within the ED when discharging older adults

Can you tell me about the process when an older person is discharged from the ED during “normal” hours?

Prompt-is there a Frailty Intervention Therapy Team (FITT) in your ED and how long has it been in place/established?

If so, what is in place? What does the FITT team involve? Who are the members? What hours do they work?

What are the specific roles (GP Liaison nurse in the ED for Older People or other role, porter) of staff in your department who are involved with older people discharge?

Who contacts the PHN and when/how is this done?

Who communicates with the GP-how and when is this performed?

What if any risk assessment tools do you use for older people attending the ED e.g., VIP (Variable Indicative of Placement), Rockwood tool? How do the results of this assessment impact in the discharge of older people?

Is there a discharge checklist used in your ED, is this older person specific? Does the OOH discharge of older people be captured on this or any other documentation?

Can you tell me about the arrangements/interventions for the out of hours discharge of older people from the ED? Prompt: If an older person has been discharged from your ED out of hours, do they remain in ED overnight for assessment/referral until the following morning? Are you aware of any issues that have arisen when discharging older people out of hours?

What system is currently used in your ED for identifying the number of older people discharged out of hours from the ED? Do you feel that the number of older people can be adequately identified with the current recording system in the ED?

To what extent do you believe that the “Patient Experience Times” capture the number of older people in the emergency department?

To what extent do you feel that the experiences of discharging older people during normal hours Vs. out of hours are similar?

Can you tell me about any possible risks or concerns for older people being discharged OOH from the ED?

Could you give me an estimate of what percentage of older persons discharged out-of-hours you think require additional input from services?

To what extent do you feel there is a need to improve the experiences of older people discharged from the ED either during normal hours or out of hours? What suggestions/ ideas do you have as to how the discharge experience of older people from the ED could be developed?

Supports within the community following older person discharge from the ED

What if any follow-up of older patients discharged from your ED occurs by a staff member in the ED?

Tell me about the main health and social care personnel in the community that you can refer an older person to when discharging an older person to home from the ED?

Tell me what your recommendations are for future practice for this client group-a) within the ED/hospital when an older person is being discharged and b) in the community following discharge?

Complete the Interview

Conclude the interview if the participant has no questions or further comments.

Thank the participant for their time and cooperation.

Explain re dissemination of results of the study.
